# A Comparative Study of Machine Learning Methods for Persistence Diagrams

**DOI:** 10.3389/frai.2021.681174

**Published:** 2021-07-28

**Authors:** Danielle Barnes, Luis Polanco, Jose A. Perea

**Affiliations:** ^1^ Department of Computational Mathematics, Science and Engineering, Michigan State University, East Lansing, MI, United States; ^2^ Department of Mathematics, Michigan State University, East Lansing, MI, United States

**Keywords:** persistent homology, machine learning, topological data analysis, persistence diagrams, barcodes

## Abstract

Many and varied methods currently exist for featurization, which is the process of mapping persistence diagrams to Euclidean space, with the goal of maximally preserving structure. However, and to our knowledge, there are presently no methodical comparisons of existing approaches, nor a standardized collection of test data sets. This paper provides a comparative study of several such methods. In particular, we review, evaluate, and compare the stable multi-scale kernel, persistence landscapes, persistence images, the ring of algebraic functions, template functions, and adaptive template systems. Using these approaches for feature extraction, we apply and compare popular machine learning methods on five data sets: MNIST, Shape retrieval of non-rigid 3D Human Models (SHREC14), extracts from the Protein Classification Benchmark Collection (Protein), MPEG7 shape matching, and HAM10000 skin lesion data set. These data sets are commonly used in the above methods for featurization, and we use them to evaluate predictive utility in real-world applications.

## 1 Introduction

Persistence diagrams are an increasingly useful shape descriptor from Topological Data Analysis. One of their more popular uses to date has been as features for machine learning tasks, with success in several applications to science and engineering. Though many methods and heuristics exist for performing learning with persistence diagrams, evaluating their relative merits is still largely unexplored. Our goal here is to contribute to the comparative analysis of machine learning methods with persistence diagrams.

Starting with topological descriptors of datasets, in the form of persistence diagrams, we provide examples and methodology to create features from these diagrams to be used in machine learning algorithms. We provide the necessary background and mathematical justification for six different methods (in chronological order): the Multi-Scale Kernel, Persistence Landscapes, Persistence Images, Adcock-Carlsson Coordinates, Template Systems, and Adaptive Template Systems. To thoroughly evaluate these methods, we have researched five different data sets and the relevant methods to compute persistence diagrams from them. The datasets, persistence diagrams and code to compute the persistence diagrams is readily available for academic use.

As part of this review, we also provide a user guide for these methods, including comparisons and evaluations across the different types of datasets. After computing the six types of features, we compared the predictive accuracy of a ridge regression, random forest, and support vector machine model to assess the type of featurization that is most useful in predictive models. The code developed for this analysis is available, with some functions developed specifically for use in machine learning applications, and easy-to-use jupyter notebooks showing examples of each function with multiple dataset types.

Of these methods, Persistence Landscapes, Adcock-Carlsson Coordinates, and Template Systems are quite accurate and create features for large datasets quickly. Adaptive Template Systems and Persistence Images took somewhat longer to run, however, the Adaptive Template Systems featurization method did improve accuracy over other methods. The Multi-Scale Kernel was the most computationally intensive, and during our evaluation we did not observe instances of it outperforming other methods.

## 2 Background

**FIGURE 1:**
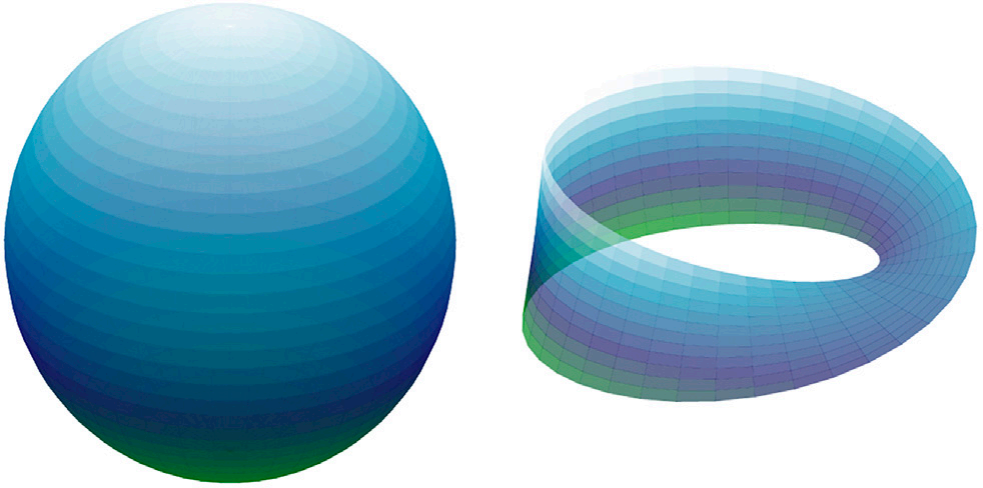
Left: The 2-dimensional sphere $S^{2}\subset {\mathbb{R}}^{3}$, right: the Möbius band ℳ.

Algebraic topology is the branch of mathematics concerned with the study of shape in abstract spaces. Its main goal is to quantify the presence of features which are invariant under continuous deformations; these include properties like the number of connected components in the space, the existence of holes and whether or not the space is orientable. As an example, Figure 1 shows two spaces: the 2-dimensional sphere on the left, which is the set $S^{2}=\left\{x\in {\mathbb{R}}^{3}:x=1\right\}$ of 3-dimensional vectors with unit norm, and the Möbius band ℳ=[−1,1]×[−1,1]/(−1,y)∼(1,−y) on the right. The latter can be thought of as the result of gluing the right and left edges of the square [−1,1]  ×  [−1,1] with opposite orientations.

The aforementioned properties of shape for these spaces are as follows. Both $S^{2}$ and ℳ are connected, while $S^{2}$ is orientable but ℳ is not. Moreover, any closed curve drawn on the surface of $S^{2}$ bounds a 2-dimensional spherical cap, and thus we say that the sphere has no 1-dimensional holes. The equator {(x,0):|x|≤1} of the Möbius band, on the other hand, is a closed curve in ℳ which is not the boundary of any 2-dimensional region, and therefore we say that ℳ has one 1-dimensional hole. Finally, $S^{2}$ is itself a closed 2-dimensional surface bounding a 3-dimensional void—thus the sphere is said to have a 2-dimensional hole—but ℳ has no such features.

The *homology* of a space is one way in which topologists have formalized measuring the presence of *n*-dimensional holes in a space (Hatcher, 2002). Indeed, for a space *X* (e.g., like the sphere or the Möbius band) an integer n≥0 and a field F (like the integers modulo a prime *p*, denoted ${\mathbb{Z}}_{p}$), the *n*-th homology of *X* with coefficients in F is a vector space over F denoted $H_{n}\left(X;F\right)$. The main point is that the dimension of this vector space corresponds roughly to the number of essentially distinct *n*-dimensional holes in *X*. Going back to the examples from Figure 1:

$$\begin{array}{ccc} H_{0}\left(S^{2};{\mathbb{Z}}_{2}\right)={\mathbb{Z}}_{2} & , & H_{0}\left(ℳ;{\mathbb{Z}}_{2}\right)={\mathbb{Z}}_{2}\\ H_{1}\left(S^{2};{\mathbb{Z}}_{2}\right)=0 & , & H_{1}\left(ℳ;{\mathbb{Z}}_{2}\right)={\mathbb{Z}}_{2}\\ H_{2}\left(S^{2};{\mathbb{Z}}_{2}\right)={\mathbb{Z}}_{2} & , & H_{2}\left(ℳ;{\mathbb{Z}}_{2}\right)=0 \end{array}$$

where, again, the dimension of $H_{0}\left(X;F\right)$ corresponds to the number of connected components in *X*, the dimension of $H_{1}\left(X;F\right)$ represents the number of 1-dimensional holes, and so on for $H_{n}\left(X;F\right)$ and n  ≥  1. It is entirely possible that different choices of F result in different dimensions for $H_{n}\left(X;F\right)$; this is an indication of intricate topological structure in *X*, but the metaphor of holes is still useful.

### 2.1 Persistent Homology

There are several learning tasks where each point in a data set has shape or geometric information relevant to the problem at hand. Indeed, in shape retrieval, database elements are often 3D meshes discretizing physical objects, and the ensuing learning tasks are often related to pose-invariant classification (Pickup et al., 2014). In computational chemistry and drug design, databases of chemical compounds are mined in order to discover new targets with desirable functional properties. In this case, the shape of each molecule (i.e., of the collection of comprising atoms) is closely related to molecular function, and thus shape features can be useful in said data analysis tasks (Bai et al., 2009).

If homology is what topologists use to measure the shape of abstract spaces, then *persistent homology* is how the shape of a geometric data set can be quantified (Perea, 2019). Persistent homology takes as input an increasing sequence of spaces

$$X:X_{0}\subset X_{1}\subset \cdots \subset X_{L}.$$

Any such sequence is called a *filtration*. The definition of persistent homology relies on two facts: first, that one can compute homology for each space separately, i.e., $H_{n}\left(X_{\ell };F\right)$ for each 0≤ℓ≤L, and second, that each inclusion $X_{\ell }\subset X_{\ell +1}$ induces a linear transformation $H_{n}\left(X_{\ell };F\right)\to H_{n}\left(X_{\ell +1};F\right)$ between the corresponding vector spaces. The *n*-th persistent homology of the filtration X is the sequence

$$PH_{n}\left(X;F\right):H_{n}\left(X_{0};F\right)\to H_{n}\left(X_{1};F\right)\to \cdots \to H_{n}\left(X_{L};F\right)$$

of vector spaces and induced linear transformations.

The evolution of features in $PH_{n}\left(X;F\right)$, which is the main point of interest, can be encoded and visualized through a *persistence diagram*. In a nutshell, if each $H_{n}\left(X_{j};F\right)$ is finite dimensional and $\beta_{n}^{j,\ell }\left(X;F\right)$ denotes the rank of the linear transformation $H_{n}\left(X_{j};F\right)\to H_{n}\left(X_{\ell };F\right)$ induced by the inclusion $X_{j}\subset X_{\ell }$, j≤ℓ, then the persistence diagram of $PH_{n}\left(X;F\right)$, denoted $\mathrm{dg}m_{n}\left(X;F\right)$, is the collection of pairs (j,ℓ) with nonzero multiplicity (i.e., number of repeats).

$$\mu_{n}^{j,\ell }:=\beta_{n}^{j,\ell -1}\left(X;F\right)-\beta_{n}^{j-1,\ell -1}\left(X;F\right)-\beta_{n}^{j,\ell }\left(X;F\right)+\beta_{n}^{j-1,\ell }\left(X;F\right),\;0\leq j<\ell \leq L.$$

See section VII.1 of Edelsbrunner and Harer (2010) for more details. In other words, $\mathrm{dg}m_{n}\left(X;F\right)$ is a multiset (i.e., a set whose elements appear with multiplicity) of pairs, where each $\left(j,\ell \right)\in \mathrm{dg}m_{n}\left(X;F\right)$ encodes $\mu_{n}^{j,\ell }$ homological features of the filtration X which appear at $X_{j}$ (i.e., *j* is the *birth time*) and disappear entering $X_{\ell }$ (ℓ is the *death time*). The *persistence* ℓ−j of (j,ℓ) is often used as a measure of prominence across the filtration X, but short-lived features can be quite informative for learning purposes as well [see for instance Bendich et al. (2016)].

#### 2.1.1 Filtrations From Point Cloud Data

**FIGURE 2:**
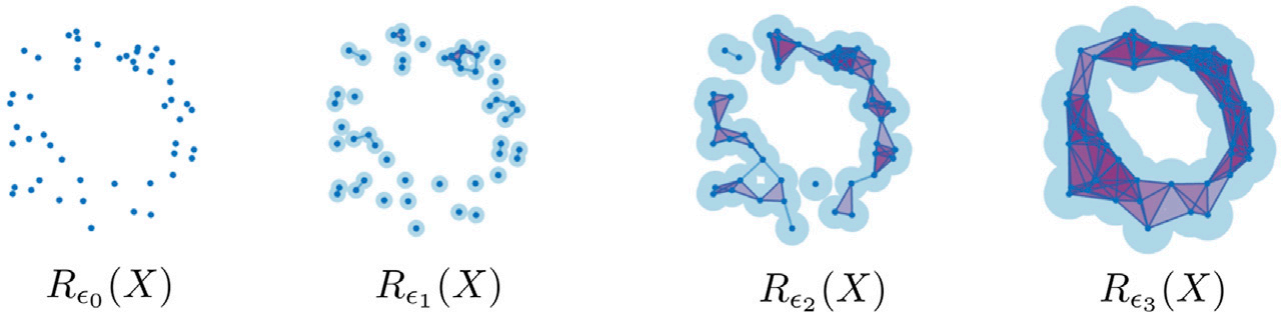
The Rips complex on a point cloud $\left(X,d_{X}\right)$ sampled around the unit circle, for four different scale choices.

There are several ways of constructing filtrations from geometric data. Indeed, let *X* be a set and $d_{X}$ a measure of distance between its elements. The pair $\left(X,d_{X}\right)$ is often referred to as point cloud data, and the running hypothesis is that it is the result of sampling *X* from an unknown continuous space. The ensuing inference problem in Topological Data Analysis is to use $\left(X,d_{X}\right)$ to estimate shape/homological features of the unknown underlying space. A popular strategy is to compute the *Vietoris-Rips complex*

$$R_{\varepsilon }\left(X\right):=\left\{\left\{x_{0},\ldots ,x_{m}\right\}\subset X|\underset{0\leq j,k\leq m}{\max}d_{X}\left(x_{j},x_{k}\right)\leq \varepsilon ,m\in \mathbb{N}\right\}$$ (1)

where ϵ≥0, a singleton {x} is thought of as a vertex at *x*, a set with two elements $\left\{x_{0},x_{1}\right\}$ represents an edge between $x_{0}$ and $x_{1}$, a set $\left\{x_{0},x_{1},x_{2}\right\}$ spans a triangle, and so on. This construction is motivated by the fact that $R_{\epsilon }\left(X\right)$ is known to approximate the topology of the underlying space from which *X* was sampled under various conditions on *X* and *ϵ* (Latschev, 2001). In practice, however, an optimal choice of scale ϵ≥0 is unclear at best, so one instead considers the *Vietoris-Rips filtration*

$$ℛ\left(X\right):R_{\varepsilon_{0}}\left(X\right)\subset R_{\varepsilon_{1}}\left(X\right)\subset \cdots \subset R_{\varepsilon_{L}}\left(X\right)$$ (2)

for $0\leq \epsilon_{0}<\epsilon_{1}<\cdots <\epsilon_{L}$. The $\epsilon_{\ell }$’s can be chosen, for instance, to be the different values of the distance function $d_{X}$. Figure 2 shows an example of this construction for X⊂ℂ sampled around the unit circle $S^{1}=\left\{z\in \mathbb{C}:\left|z\right|=1\right\}$, and four scales ϵ≥0.

**FIGURE 3:**
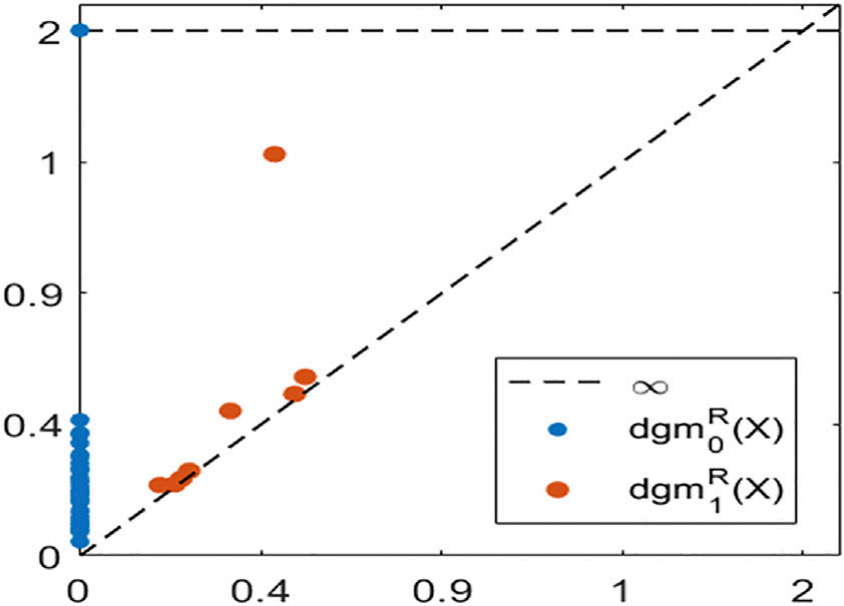
The Rips persistence diagrams in dimensions 0 (blue) and 1 (orange), for a point cloud sampled around the unit circle.

The persistent homology of the Vietoris-Rips filtration, i.e., $PH_{n}\left(ℛ\left(X\right);F\right)$, can then be used to measure the shape of the underlying shape of the point cloud. An important point is that even though homology is invariant under continuous deformations, the Vietoris-Rips complex is a metric-based construction. Thus, the resulting Vietoris-Rips persistence diagrams

$$\mathrm{dg}m_{n}^{ℛ}\left(X\right)=\left\{\left(\epsilon_{j},\epsilon_{\ell }\right)\text{with}\;\text{multiplicity}\;\mu_{n}^{j,\ell }>0\right\}$$

often encode features such as density and curvature, in addition to the presence of holes and voids (Bubenik et al., 2020). Figure 3 shows the Vietoris-Rips persistence diagrams in dimensions n=0,1 for the data sampled around the unit circle in Figure 2. The persistence of a point in a persistence diagram can be visualized as its vertical distance to the diagonal. This measures how likely it is for said feature to correspond to one of the underlying space, instead of being a reflection of sampling artifacts [see for instance Theorem 5.3 in Oudot (2015)]. The fact that there is one highly persistent point for n=0 indicates that the data has one cluster (i.e., one connected component), while the presence of one highly persistent point for n=1 indicates that there is a strong 1-dimensional hole in the data. Both are consistent with, and suggest, that the circle is the underlying space.

#### 2.1.2 Filtrations From Scalar Functions and Image Data

**FIGURE 4:**
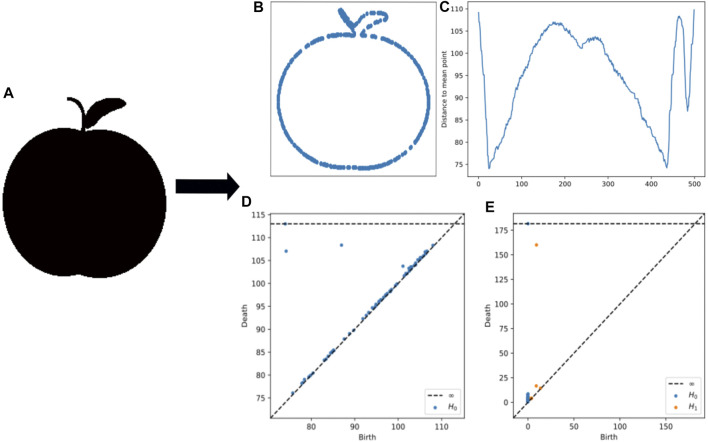
**(A)** An example apple from MPEG7 data. **(B)** An example image contour used for MPEG7. **(C)** The distance to mean point calculation used for sublevel set persistence **(D)** Persistence diagrams from lower star persistence **(E)** Persistence diagrams from the contour.

If X is a topological space and f:X→ℝ is a function, then the sublevel sets

$$X_{a}=f^{-1}\left(-\infty ,a\right],\;a\in \mathbb{R}$$

define the so called *sublevel set filtration* of X. If X is a 3D mesh, for example, then one can compute estimates of curvature at every vertex, and then extend said function linearly (*via* barycentric coordinates) to the triangular faces. The persistent homology of the sublevel set filtration is often called *sublevel set persistence*, and it is useful in quantifying shape properties of geometric objects which are endowed with scalar functions. See Figure 4 for an application of this idea. The corresponding persistence diagrams are denoted $\mathrm{dg}m_{n}\left(f\right)$.

**FIGURE 5:**
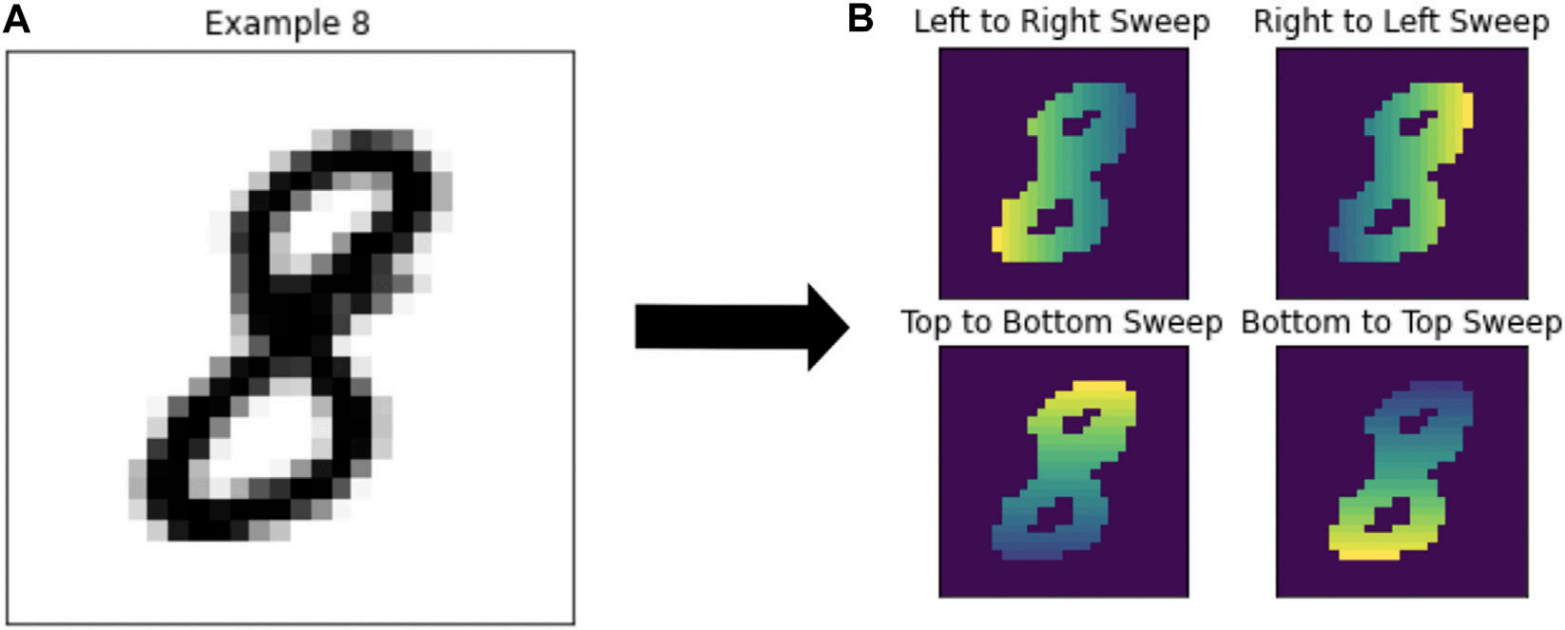
**(A)** Example number 8 from the MNIST dataset. **(B)** The same number 8 after computing each of the four types of coordinate transforms to compute the persistence diagrams.

Images provide another data modality where sublevel set persistence can be useful. Indeed, an image can be thought of as a function on a grid of pixels; if the image is in grey scale, then we have a single scalar valued function, and if the image is multi-channel (like RGB color representations) then each channel is analyzed independently. The grid yields a triangulated space *via* a Freudenthal triangulation of the plane, and the values of pixel intensity in each channel can be extended *via* convex combinations to the faces [see Lemma 1 of Kuhn (1960)]. We will apply this methodology later on to the MNIST hand written digit data base (Figure 5). This approach to computing persistent homology from images is not unique in the literature; other popular methods such as cubical homology (Kaczynski et al., 2004) have been used for this same purpose. This work, however, deals exclusively with simplicial homology as it is the standard approach in many applications.

### 2.2 The Space of Persistence Diagrams

Persistence diagrams have shown to be a powerful tool for quantifying shape in geometric data (Carlsson, 2014). Moreover, one of their key properties is their stability with respect to perturbations in the input, which is crucial when dealing with noisy measurements. Indeed, two persistence diagrams *D* and $D^{\prime }$ are said to be *δ*-matched, δ>0, if there exists a bijection $m:A\to A^{\prime }$ of multisets A⊂D and $A^{\prime }\subset D^{\prime }$ with.

${\left\|x-m\left(x\right)\right\|}_{\infty }<\delta$ for every x∈A, where $∥\cdot {∥}_{\infty }$ is the maximum metric in ${\mathbb{R}}^{2}$.
If $\left(a,b\right)\in \left(D\setminus A\right)\cup \left(D^{\prime }\setminus A^{\prime }\right)$, then b−a<2δ.

The *bottleneck distance* $d_{B}\left(D,D^{\prime }\right)$ is the infimum over all δ>0 so that *D* and $D^{\prime }$ are *δ*-matched; this defines a metric on the set $D_{0}$ of all finite persistence diagrams. The *stability* theorem of Cohen-Steiner et al. (2007) for sublevel set persistence contends that if X is a finitely triangulated space and f,g:X→ℝ are *tame* and continuous, then

$$d_{B}\left(\mathrm{dg}m_{n}\left(f\right),\mathrm{dg}m_{n}\left(g\right)\right)\leq {\left\|f-g\right\|}_{\infty }$$

for every integer n≥0. We note that the theorem is still true if continuous is replaced by piecewise linear. Similarly, if $\left(X,d_{X}\right)$ and $\left(Y,d_{Y}\right)$ are finite metric spaces, then the stability of Rips persistent homology (Chazal et al., 2014, Theorem 5.2) says that

$$d_{B}\left(\mathrm{dg}m_{n}^{ℛ}\left(X\right),\mathrm{dg}m_{n}^{ℛ}\left(Y\right)\right)\leq 2d_{GH}\left(X,Y\right)$$

where $d_{GH}\left(\cdot ,\cdot \right)$ denotes the Gromov-Hausdorff distance (Gromov, 2007).

In order to develop the mathematical foundations needed for doing machine learning with persistence diagrams, it has been informative to first study the structure of the space they form. Indeed, if $D_{0}$ denotes the space of finite persistence diagrams, then we will let D denote its metric completion with respect to the bottleneck distance $d_{B}$. It readily follows that $d_{B}$ extends to a metric on D. See Blumberg et al. (2014) for an explicit description of what the elements of D are. In addition to the bottleneck distance, the *Wasserstein metric* from optimal transport suggests another way of measuring similarity between persistence diagrams. Indeed, for each integer p≥1 and $D,D^{\prime }\in D$, their *p*-th Wasserstein distance is

$$d_{W_{p}}\left(D,D^{\prime }\right):=\underset{m}{\inf}{\left(\underset{x\in A}{\sum }{\left|\left|x-m\left(x\right)\right|\right|}_{\infty }^{p}+\underset{\left(a,b\right)\in \left(D\setminus A\right)\cup \left(D’\setminus A’\right)}{\sum }{\left(\frac{b-a}{2}\right)}^{p}\right)}^{1/p}$$

where the infimum runs over all multiset bijections $m:A\to A^{\prime }$, for A⊂D and $A^{\prime }\subset D^{\prime }$. One can show that $d_{W_{p}}$ defines a metric on the set

$$D_{p}:=\left\{D\in D|d_{W_{p}}\left(D,\emptyset \right)<\infty \right\}$$

and that $\left(D_{p},d_{W_{p}}\right)$ is a complete separable metric space (Mileyko et al., 2011) with $d_{W_{p}}\to d_{B}$ as p→∞.

Doing statistics and machine learning directly on the space of persistence diagrams turns out to be quite difficult. Indeed, $\left(D,d_{B}\right)$ does not have unique geodesics, and thus the Fréchet mean of general collections of persistence diagrams is not unique (Turner et al., 2014). Since computing averages, and in general, doing linear algebra on persistence diagrams is not available, then several authors have proposed mapping $\left(D,d_{B}\right)$ to topological vector spaces where further analysis can be done. These methods are the main focus of this review. The theory of vectorization of persistence diagrams is an active area of research, with recent results showing the impossibility of full embeddability. Indeed, even though the space of persistence diagrams with exactly *n* points can be coarsely embedded in a Hilbert space (Mitra and Virk, 2021), this ceases to be true if the number of points is allowed do vary (Wagner, 2019; Bubenik and Wagner, 2020). That said, partial featurization is still useful as we will demonstrate here.

## 3 Featurization Methods

For each of the methods below, we start with a collection of persistence diagrams. A persistence diagram can be represented in either the birth-death plane or birth-lifetime plane—some methods will require birth-death coordinates and others will require birth-lifetime coordinates. The **birth-death plane** is the representation pair (x,y) where *x* is the time of birth, and *y* is the time of death of the feature in the persistence diagram. The **birth-lifetime plane** can be defined as the collection of points (x,y−x), where (x,y) is in birth-death coordinates. In this manner, we define lifetime as the persistence y−x of a feature (x,y). The persistence diagrams of a particular geometric object can be calculated in a variety of ways, which will be made explicit for each dataset at time of evaluation.

### 3.1 Multi-Scale Kernel

The Multi-Scale Kernel of Reininghaus et al. (2015) defines a Kernel over the space of persistence diagrams, which can then be used in various types of kernel learning methods. In general, a kernel *k* is by definition a symmetric and positive definite function of two variables. Mathematically, from Reininghaus et al. (2015), given a set *X*, a function k:X×X→ℝ is a *kernel* if there exists a Hilbert space *H*, called the *feature space*, and a map Φ:X→H, called the *feature map*, such that $k\left(x,y\right)={\langle \Phi \left(x\right),\Phi \left(y\right)\rangle }_{H}$ for all x,y∈X. The kernel induces a distance on *X* defined as

$$d_{k}\left(x,y\right)={\left(k\left(x,x\right)+k\left(y,y\right)-2k\left(x,y\right)\right)}^{\frac{1}{2}}={\left|\left|\Phi \left(x\right)-\Phi \left(y\right)\right|\right|}_{H}.$$

Reininghaus et al. (2015) propose a multi-scale kernel on D as follows. Given F,G∈D, the persistence scale space kernel $k_{\sigma }$ is

$$k_{\sigma }\left(F,G\right)={\langle \Phi_{\sigma }\left(F\right),\Phi_{\sigma }\left(G\right)\rangle }_{L^{2}\left(\Omega \right)}$$ (3)

where $\Phi_{\sigma }:D\to L^{2}\left(\Omega \right)$ is the associated feature map, and $\text{Ω}\subset {\mathbb{R}}^{2}$ is the closed half-plane above the diagonal. Deriving the solution of a distribution-analogue of the Heat equation with boundary conditions in Definition 1 of Reininghaus et al. (2015), the closed form expression of the multi-scale kernel is:

$$k_{\sigma }\left(F,G\right)=\frac{1}{8\pi \sigma }\sum_{p\in F,q\in G}e^{-\frac{{\left|\left|p-q\right|\right|}^{2}}{8\sigma }}-e^{-\frac{p-{\bar{q}}^{2}}{8\sigma }}$$

where if q=(a,b), then $\bar{q}=\left(b,a\right)$.

The multi-scale kernel is shown to be stable w.r.t the 1-Wasserstein distance by Theorem 2 of Reininghaus et al. (2015), which is a desirable property for classification algorithms. However, by Theorem 3 of Reininghaus et al. (2015), the multi-scale kernel is not stable in the Wasserstein sense for 1<p≤∞.

### 3.2 Persistence Landscapes

Persistence landscapes are a mapping of persistence diagrams into a function space that is either a Banach space or Hilbert space (Bubenik, 2020). Advantages of persistence landscapes are that they are invertible, stable, parameter-free, and nonlinear. Persistence landscapes can be computed from a persistence diagram as follows.

From Bubenik (2020), for a persistence diagram $D={\left(a_{i},b_{i}\right)}_{i\in I}$, and for a<b, let

$$f_{\left(a,b\right)}\left(t\right)=\max\left(0,\min\left(a+t,b-t\right)\right)$$ (4)

and

$$\lambda_{k}\left(t\right)=\text{kmax}{\left\{f_{\left(a_{i},b_{i}\right)}\left(t\right)\right\}}_{i\in I}$$ (5)

with kmax as the kth largest element.

**FIGURE 6:**
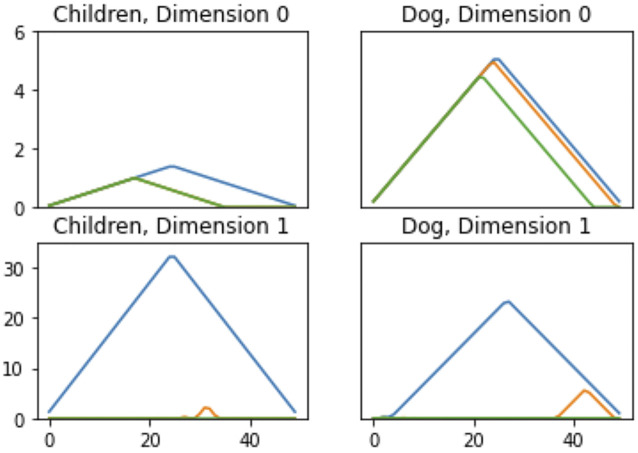
Persistence Landscapes from the MPEG7 dataset to show differences in features. Each color corresponds to a different landscape, i.e., $\lambda_{k}$ for k=1,2,3.

The *persistence landscape* is the sequence of piecewise linear functions, $\lambda_{1},\lambda_{2},\ldots :\mathbb{R}\to \mathbb{R}$. Bubenik shows desirable properties for working with persistence landscapes in statistical modeling, in particular that even if unique means do not exist in the set of persistence diagrams, persistence landscapes do have unique means and the mean landscape converges to the expected persistence landscape. Figure 6 shows an example of persistence landscapes from the MPEG7 dataset, described in the data section.

### 3.3 Persistence Images

From Adams et al. (2015), persistence images are a mapping sending a persistence diagram to an integrable function, called a persistence surface. Fixing a grid on ${\mathbb{R}}^{2}$, the integral over this grid yields pixel values forming the persistence image. Advantages of persistence images include a representation in ${\mathbb{R}}^{n}$, stability, and ease of computation. When calculating the persistence image, a resolution, a distribution, and a weighting function are required as parameters. It is worth noting that the resolution (i.e., number of pixels) determines the number of features computed by the persistence image.

More explicitly, let *D* be a persistence diagram in birth-lifetime coordinates. We take $\phi_{u}:{\mathbb{R}}^{2}\to \mathbb{R}$ to be a differentiable probability distribution. Using, for instance, the Gaussian Distribution with mean *u* and variance $\sigma^{2}$ we have

$$\phi_{u}\left(x,y\right)=\frac{1}{2\pi \sigma^{2}}e^{-\left[{\left(x-u_{x}\right)}^{2}+{\left(y-u_{y}\right)}^{2}\right]/2\sigma^{2}}$$

**FIGURE 7:**
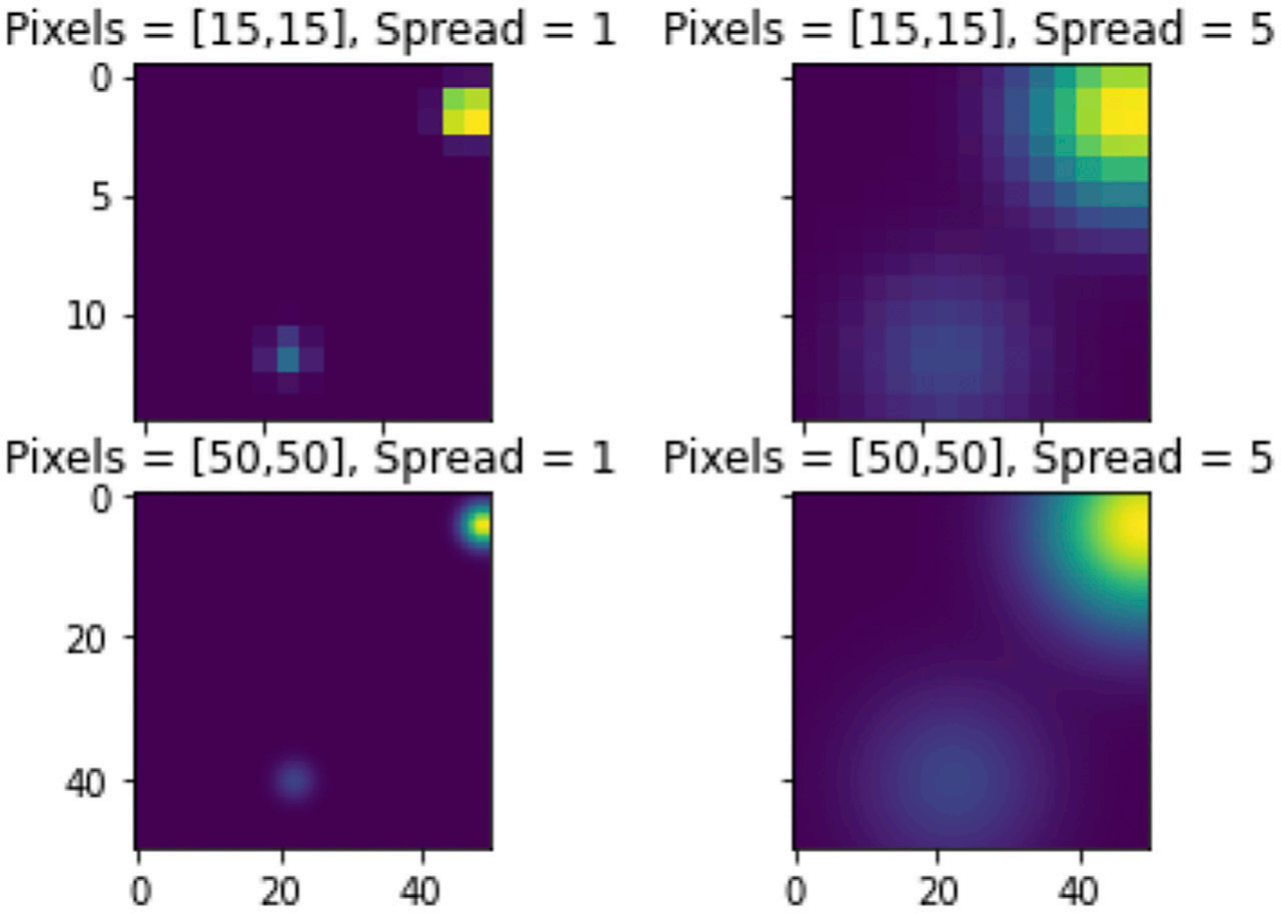
Persistence Images of a 5 from the MNIST set in dimension 0.

The persistence surface $\rho_{D}:{\mathbb{R}}^{2}\to \mathbb{R}$ is the function

$$\rho_{D}\left(z\right)=\underset{u=\left(x,y-x\right)\in D}{\sum }f\left(u\right)\phi_{u}\left(z\right)$$

with $f:{\mathbb{R}}^{2}\to \mathbb{R}$, a nonnegative weighting function that is zero along the horizontal axis, continuous, and piecewise differentiable. The **persistence image** is then $I\left(\rho_{D}\right)p=\iint_{p}\rho_{D}dydx$, where integration is over the fixed grid on ${\mathbb{R}}^{2}$. This creates an image depicting high and low density areas in the defined grid, that are represented as a high-dimensional vector for use in machine learning algorithms. An example is shown in Figure 7 taken from the MNIST dataset.

### 3.4 Adcock-Carlsson Coordinates: The Ring of Algebraic Functions on Persistence Diagrams

This method is explored by Adcock et al. (2016) where the authors highlight the fact that any persistence diagram with exactly *n* points can be described by a vector of the form $\left(x_{1},y_{1},x_{2},y_{2},\ldots ,x_{n},y_{n}\right)$ where $x_{i}$ denotes the birth of the *i*-th class and $y_{i}$ the corresponding death time. Since this specific representation imposes an arbitrary ordering of the elements in the persistence diagram, one can more precisely identify the set of persistence diagrams with exactly *n* points with elements of the *n*-symmetric product of ${\mathbb{R}}^{2}$, denoted $Sp^{n}\left({\mathbb{R}}^{2}\right)$.

The inclusions $Sp^{n}\left({\mathbb{R}}^{2}\right)↬Sp^{n+1}\left({\mathbb{R}}^{2}\right)$ thus produce an inverse system of affine coordinate rings

$$\cdots \to A\left[Sp^{n+1}\left({\mathbb{R}}^{2}\right)\right]\to A\left[Sp^{n}\left({\mathbb{R}}^{2}\right)\right]\to \cdots$$

which provide the basis for studying algebraic functions on the space of persistence diagrams.

With this setting in mind, the main goal of Adcock et al. (2016) is to determine free generating sets for the subalgebra of $A\left[Sp^{\infty }\left({\mathbb{R}}^{2}\right)\right]$ comprised of elements which are invariant under adjoining a point of zero persistence to a persistence diagram. The following theorem is an answer to this question (see Theorem 1 Adcock et al. (2016)).

Theorem 1 The subalgebra of 2-multisymmetric functions invariant under adding points with zero persistence, is freely generated over ℝ by the set of elements of the form

$$p_{a,b}=\underset{i}{\sum }{\left(x_{i}+y_{i}\right)}^{a}{\left(y_{i}-x_{i}\right)}^{b}$$

for integers a≥0 and b≥1.

These are the features we call *Adcock-Carlsson coordinates*.

Using this method we chose the following features for both the 0-dimensional and 1-dimensional persistence diagrams, as suggested in Adcock et al. (2016) when analyzing the MNIST data set: $\underset{i}{\sum }x_{i}\left(y_{i}-x_{i}\right),\underset{i}{\sum }\left(y_{\max}-y_{i}\right)\left(y_{i}-x_{i}\right),\underset{i}{\sum }x_{i}^{2}{\left(y_{i}-x_{i}\right)}^{4},\underset{i}{\sum }{\left(y_{\max}-y_{i}\right)}^{2}{\left(y_{i}-x_{i}\right)}^{4}$.

### 3.5 Template Systems

The goal of this method is to find features for persistence diagrams by finding dense subsets of C(D,ℝ). To accomplish this we will rely on the fact that given a persistence diagram D∈D, and a continuous and compactly supported real-valued function on $W=\left\{\left(x,y\right)\in {\mathbb{R}}^{2}:0\leq x<y\right\}$, i.e. for $f\in C_{c}\left(W\right)$, we can define a continuous [see Theorem 26 Perea et al. (2019)] map $\nu \left(D\right):C_{c}\left(W\right)\to \mathbb{R}$ given by

$$\nu \left(D,f\right):=\sum_{x\in D}f\left(x\right).$$

The function D↦ν(D,⋅) defines a continuous injection $D↬C_{c}{\left(W\right)}^{\prime }$ into the topological dual of $C_{c}\left(W\right)$. The specific topology in the codomain is chosen so that *ν* is in fact continuous.

This injective featurization allows us to define a **template system** for D as a collection $T\in C_{c}\left(W\right)$ such that ${ℱ}_{T}:=\left\{\nu \left(\cdot ,f\right):D\to \mathbb{R}|f\in T\right\}$ separates points. That is, if $D,D^{\prime }\in D$ are distinct, then there exists f∈T for which $\nu \left(D,f\right)\neq \nu \left(D^{\prime },f\right)$.

The advantage of working with these template systems is that they can be used to approximate real-valued functions on the space of persistence diagrams as proven by the following theorem [see Theorem 29 Perea et al. (2019)].

Theorem 2 Let $T\subset C_{c}\left(W\right)$ Be a Template System for D, let C⊂D Be Compact, and let F:C→ℝ Be Continuous. Then for Every ϵ>0 There Exist N∈ℕ, a Polynomial $p\in \mathbb{R}\left[x_{1},\ldots ,x_{N}\right]$ and Template Functions $f_{1},\ldots ,f_{N}\in T$ so That

$$\left|p\left(\nu \left(D,f_{1}\right),\ldots ,\nu \left(D,f_{N}\right)\right)-F\left(D\right)\right|<\varepsilon$$

for every D∈C.

That is, the collection of functions of the form $D\to p\left(\nu \left(D,f_{1}\right),\ldots ,\nu \left(D,f_{N}\right)\right)$, is dense in C(D,ℝ) with respect to the compact-open topology.

Even though this theorem provides the theoretical underpinnings to guarantee the existence of solutions to supervised machine learning problems, it does not provide the specifics for doing so. In particular, one question to answer is how to choose suitable families of template functions. In our evaluations we will explore both prescribed families for template systems, as well as data-driven or adaptive ones.

**FIGURE 8:**
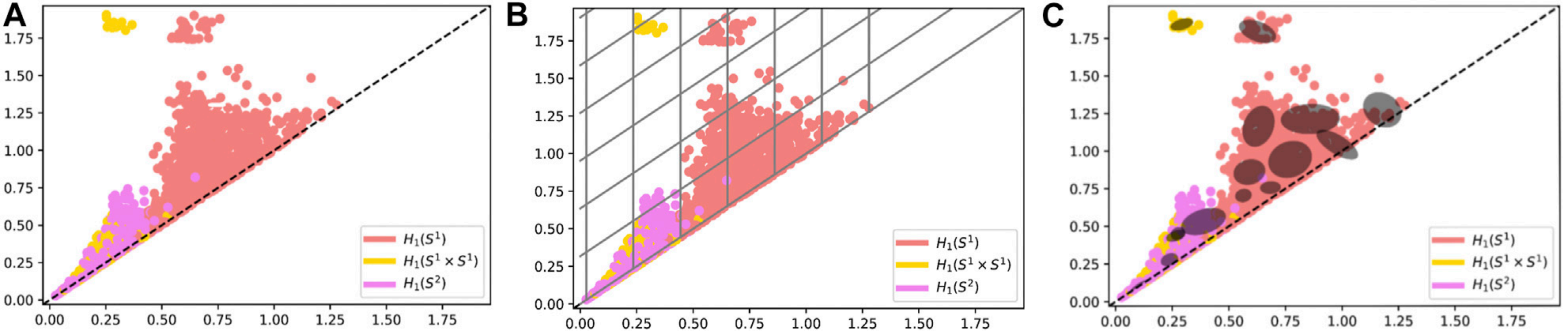
**(A)** Persistence Diagrams **(B)** Persistence Diagrams with boundaries of the support function for Tent Features **(C)** Persistence Diagrams with boundaries of the support for Adaptive Templates.

In the prescribed front we have the tent functions described below. See also Figure 8. In the birth-lifetime plane, and given a point x=(a,b)∈W and a discretization scale 0<δ<b, the associated tent function on W is given by

$$g_{x,\delta }\left(x,y\right)={\left|1-\frac{1}{\delta }\max\left\{\left|x-a\right|,\left|y-b\right|\right\}\right|}_{+}$$

where ${\left|r\right|}_{+}=\max\left\{0,r\right\}$. As δ<b, this function has support in the compact box [a−δ,a+δ]×[b−δ,b+δ]⊂W. Given a persistence diagram D∈D in birth-death coordinates, the value of the tent function is

$$G_{x,\delta }\left(D\right)=\underset{\left(x,y\right)\in D}{\sum }g_{x,\delta }\left(x,y-x\right).$$

### 3.6 Adaptive Template Systems

The Adaptive Template Systems methodology of Polanco and Perea (2019a) concerns itself with improving and furthering some of the work presented in Perea et al. (2019). The goal is to produce template systems that are attuned or adaptive to the input data set and the supervised classification problem at hand. One shortcoming of template systems, like tent functions, when applied to Theorem 2 is that without prior knowledge about the compact set C⊂D, the number of template functions that carry no information relevant to the problem can be high. By reducing this overhead, adaptive templates improve the computation times and accuracy in some specific problems.

The relationship between template systems and adaptive template systems is demonstrated in Figure 4, showing the adaptive template systems depend on density of data. To do so, given a compact set C⊂D we consider the set $S=\underset{D\in C}{\cup }D\subset W$ along with different algorithms such as Gaussian mixture models (GMM) (Reynolds, 2009), Hierarchical density-based spatial clustering of applications with noise (HDBSCAN) (Campello et al. ,2013) and Cover-Tree Entropy Reduction (CDER) (Smith et al., 2017) to define a family of ellipsoidal domains $\left\{z\in {\mathbb{R}}^{2}:{\left(z-x\right)}^{*}A\left(z-x\right)\leq 1\right\}$ in W, fitting the density distribution of *S*. Here *A* is a 2×2 symmetric matrix and $x\in {\mathbb{R}}^{2}$.

Once this family of ellipsoidal domains is computed, we use them to define the following adaptive template functions

$$f_{A}\left(z\right)=\{\begin{array}{ll} 1-{\left(z-x\right)}^{*}A\left(z-x\right) & \mathrm{if}\;{\left(z-x\right)}^{*}A\left(z-x\right)<1\\ 0 & \mathrm{if}\;{\left(z-x\right)}^{*}A\left(z-x\right)\geq 1 \end{array}$$

### 3.7 Other Approaches

The featurization methods presented in this section are by no means an exhaustive list of what is available in the literature. Here are some others that the interested reader may find useful:

• The **Persistent homology rank functions** of Robins and Turner (2016) are similar in spirit to persistent landscapes, in that they provide an injective inclusion of $D_{0}$ into a Hilbert space of functions where techniques like functional Principal Component Analysis are available. Indeed, for a filtration X, its *n*-th persistent rank function is defined as

$$\begin{array}{lll} W & \to & \mathbb{R}\\ \left(a,b\right) & \mapsto & \beta_{n}^{a,b}\left(X\right)=\mathrm{rank}\left(H_{n}\left(X_{a}\right)\to H_{n}\left(X_{b}\right)\right). \end{array}$$

This is equivalent, for a persistence diagram $D\in D_{0}$, to defining the function

$$\begin{array}{lll} W & \to & \mathbb{R}\\ \left(a,b\right) & \mapsto & \#\left\{\left(x,y\right)\in D:x\leq a\;\mathrm{and}\;y>b\right\} \end{array}$$

where # is multiset cardinality. The Hilbert space in question is the weighted $L^{2}$-space $L^{2}\left(W,\phi \right)$. Here ϕ:[0,∞)→[0,∞) satisfies $\int_{0}^{\infty }\phi \left(t\right)dt<\infty$, and the inner product of rank functions is

$${\langle f,g\rangle }_{\phi }=\int_{W}f\left(x,y\right)g\left(x,y\right)\phi \left(y-x\right)dxdy.$$

This approach has shown to be effective in analyzing point processes, and sphere packing patterns.

The **Persistent curve** (Chung et al., 2018; Giusti et al., 2015) provides another functional summary closely related to persistent rank functions. Specifically, for a persistence diagram $D\in D_{0}$, its persistence curve (Chung et al., 2018) is the function

$$\begin{array}{lll} \left[0,\infty \right) & \to & \left[0,\infty \right)\\ t & \mapsto & \#\left\{\left(x,y\right)\in D:x\leq t<y\right\}. \end{array}$$

Discretizations of these curves have been useful in computer vision tasks (Chung and Lawson, 2020), as well as in neuroscience applications (Giusti et al., 2015).

Other **kernel methods**, besides the Multi-Scale kernel of Reininghaus et al. (2015), have appeared in the literature. They correspond to the following choices of kernel function $k:D_{0}\times D_{0}\to \mathbb{R}$. The *Persistence Weighted Gaussian Kernel* of Kusano et al. (2016) is defined as

$$k_{PWG}\left(D,D\prime \right)=\sum_{\begin{array}{l} x=\left(x,y\right)\in D\\ x\prime =\left(x^{\prime },y^{\prime }\right)\in D^{\prime } \end{array}}\arctan\left(C|y-x{|}^{p}\right)\cdot \arctan\left(C|y^{\prime }-x^{\prime }{|}^{p}\right)\cdot e^{-\frac{{\left|\left|x-x\prime \right|\right|}^{2}}{2\sigma^{2}}}$$

for parameters C,p,σ>0, while the *Sliced Wassertein Kernel* of Carriere et al. (2017) takes the form

$$k_{SW}\left(D,D^{\prime }\right)=\exp^{\frac{-d_{SW}\left(D,D^{\prime }\right)}{2\sigma^{2}}}$$

where $d_{SW}\left(\cdot ,\cdot \right)$ is the so-called sliced Wasserstein distance between persistence diagrams [see Eq. 2 and Definition 3.1 of Carriere et al. (2017)]. If instead one uses the Fisher Information metric $d_{FIM}\left(\cdot ,\cdot \right)$ [see Eq. 3 of Le and Yamada (2018)], then the result is the *Persistence Fisher Kernel*

$$k_{PF}\left(D,D^{\prime }\right)=e^{-td_{FIM}\left(D,D^{\prime }\right)},t>0.$$

Persistence diagrams as features for **deep neural networks** have also been studied recently. In particular, the *PersLay* framework of Carrière et al. (2020) leverages the Deep Sets architecture of Zaheer et al. (2017) to implement layers that can process persistence diagrams. Specifically, layers of the form:

$$D\mapsto \mathrm{op}\left({\left\{\omega \left(x\right)\phi \left(x\right)\right\}}_{x\in D}\right)$$

where op(⋅) is a permutation invariant operator (e.g., max, min, sum, etc), $\omega :{\mathbb{R}}^{2}\to \mathbb{R}$ is a weight function, and $\phi :{\mathbb{R}}^{2}\to {\mathbb{R}}^{q}$ is a representation function. By optimizing *ω* and *ϕ* in a parametric family—i.e., $\omega =\omega_{u}$ and $\phi =\phi_{v}$—the training of the network can lead to vectorizations attuned to specific learning tasks.

## 4 Datasets

The five different datasets considered in this work were chosen from a collection of experiments presented in the literature of topological methods for machine learning. We acknowledge that this selection is inherently bias towards datasets with favorable performance with regards to specific topological methods. Nevertheless, we counterbalance this by applying all the evaluated featurization methods to all the data sets here considered and compare the classification results across all the presented methodologies. This comparative work showcases how the variation between methods results in the need for the user to find suitable combination of featurization methods and parameter tuning to obtain optimal results in a given dataset. As such, readers should view this as a resource for their own analysis, and not as a recommendation for specific techniques.

For all datasets and methods, parameter tuning was done using a grid search method on a subset of data that was not used to report final results, and parameters were chosen based on performance of a ridge regression model, random forest and support-vector machine (SVM) model. It is worth noting a weakness of the analysis in that the same parameters were used in the feature set calculation for all reported models, and run with a single split. This was due to time required for feature calculation.

The ridge regression and random forest classifier were run with default parameters, and the support-vector machine was run using the radial basis function (RBF) with some tuning on the cost parameter (C). The exception is for the Multi-Scale Kernel feature set—we only fit a support-vector machine model. It is important to highlight that results regarding ridge regression with (polynomial and radial basis function) kernel methods are not included in this work as they produce increased computational times while the classification results do not improve significantly compared to the one presented here. Each dataset was sampled for a 10 or 100 trials depending on size, with the exception of the Protein Classification Dataset, which included indices for predefined problems.

Random forest classifiers as presented in Breiman (2001) are used to solve the same classification problems presented for each data set. Parameters such as number of trees in each forest and the size of each tree are chosen based on performance and tuned on the testing set.

### 4.1 MNIST

The MNIST dataset from LeCun and Cortes (1999) is a database of 70,000 handwritten single digits of numbers zero to nine. An example image from the MNIST database is shown in Figure 7.

The calculation of persistence diagrams for the MNIST dataset is as in Adcock et al. (2016). This method creates a base of 8 different persistence diagrams to use in the application of methods. The persistence diagrams are calculated using a “sweeping” motion in one of four directions: left to right, right to left, top to bottom, or bottom to top, corresponding to the 0-dimensional and 1-dimensional persistence diagrams. To compute this filtration, pixels are converted to a binary response with intensity calculated based on position. This has the effect that depending on the direction of sweep, features will have different birth and death times, providing distinct information for each direction. The number of topological features available for model fitting is dependent on the method. For the Persistent Images, Persistence Landscapes, and Template Systems there are eight features each. The Multi-Scale Kernel produces eight different kernel matrices, and for Adcock-Carlsson Coordinates, 32 different features were computed from these persistence diagrams.

Figure 5 shows the various calculations of persistence diagrams for an example number eight. Both 0-dimensional and 1-dimensional persistence diagrams were used for the MNIST dataset, noting that some observations did not have 1-dimensional persistence diagrams, so these observations were filled with a single diagram of birth-death coordinate of [0,.01].

For the MNIST dataset, a random sample of 1,000 images was used to tune parameters, with 80% used for the training portion, and 20% used for the testing portion. We used the set of 60,000 images corresponding to the training set of MNIST to create our own training and testing sets for model fitting and evaluation. For this set of 60,000, 10 trials were run with an 80% training and 20% testing split to determine model performance.

### 4.2 SHREC14

We evaluated the SHREC 2014 dataset (Pickup et al., 2014) in the same manner as the authors of Polanco and Perea (2019a). To compute the topological features, the authors of Reininghaus et al. (2015) describe using a heat kernel signature to compute persistence diagrams for both the 0-dimensional and 1-dimensional persistence diagrams. The dataset consists of 15 different labels, corresponding to five different poses for the three classes of male, female, and child figures.

As noted in Polanco and Perea (2019a), parameters in the dataset define different problems due to a different calculation of the heat kernel signature, and for this evaluation we focused on the problem with the highest accuracy as reported in Polanco and Perea (2019a).

For the SHREC14 dataset, a random sample of 90 images (30% of the data) was used to tune the model and determine appropriate model parameters. The remaining 210 observations were split into 80% training and 20% testing for 100 trials to report final model fit. Persistence diagrams for 0-dimensional homology and 1-dimensional homology were computed for this dataset.

Table 1 shows complete results for the SHREC 2014 dataset.

**TABLE 1:** Results from the Shrec14 Dataset using the average model classification accuracy ± standard deviation over 100 trials.

Full results for SHREC14 dataset			
Method	Train	Test	Model
Multi-scale kernel (sigma = .5, sum of kernels)	.8942±.0142	.8938±.0464	Kernel SVM
**Persistence landscapes (*n* = 5, *r* = 200)**	.9968±.0037	.9312±.0336	**Ridge regression**
	.9302±.0098	.9186±.0417	SVM (RBF, *c* = 10)
	.9739±.0190	.9114±.0441	Random forest
Persistent images (*p* = 40, *s* = .5)	.7243±.0387	.7048±.0588	Ridge regression
	.9067±.0147	.8876±.0479	SVM (RBF, *c* = 1)
	.9855±.0092	.865±.0764	Random forest
Adcock-carlsson coordinates	.85±.0199	.7124±.0814	Ridge regression
	.8671±.0183	.6928±.0599	SVM (RBF, *c* = 50)
	.9147±.0299	.6976±.0899	Random forest
Template systems (*d* = 12, *p* = 1.1)	.9442±.0087	.9100±.0405	Ridge regression
	.9350±.0079	.9159±.0383	SVM (RBF, *c* = 1)
	.9483±.0214	.8874±.0481	Random forest
**Adaptive template systems (CDER)**	.9937±.0078	.9169±.0395	**Ridge regression**
	.9929±.0083	.9064±.0397	SVM (RBF, *c* = 10)
	.9729±.0200	.9164±.0422	Random forest

### 4.3 Protein Classification

We use the Protein Classification Benchmark dataset PCB00019 Sonego et al. (2007) as another type of data to evaluate the topological methods above. This specific set contains information for 1,357 proteins corresponding to 55 classification problems, and we reported on 54 of the problems using one to tune parameters. The training and testing index were provided, and the mean accuracy was reported for both training and testing sets using these indices. Table 2 shows results from our experiments using the training and testing indices provided in the original dataset.

**TABLE 2:** Results from the Protein Dataset using the average model classification accuracy ± standard deviation over 54 trials corresponding to the predefined indices of the dataset.

Full results for protein dataset			
Method	Train	Test	Model
Multi-scale kernel (sum of kernels)	.8294±.1063	.8803±.0702	Kernel SVM
Persistence landscapes	.9108±.0615	.9620±.0204	Ridge regression
	.9012±.0682	.9782±.0151	SVM (RBF)
	.9011±.0686	.9782±.0152	Random forest
Persistent images	.9011±.0682	.9758±.0165	Ridge regression
	.9007±.0684	.9782±.0151	SVM (RBF)
	.9008±.0685	.9782±.0151	Random forest
Adcock-carlsson coordinates	.9008±.0685	.9780±.0151	Ridge regression
	.9009±.0685	.9782±.0151	SVM (RBF)
	.9015±.0677	.9779±.0151	Random forest
Template systems	.9008±.0684	.9780±.0151	Ridge regression
	.9020±.0678	.9782±.0151	SVM (RBF)
	.9016±.0678	.9775±.0152	Random forest
Adaptive template systems	.9008±.0685	.9782±.0151	Ridge regression (CDER)
	.9007±.0684	.9782±.0151	SVM (CDER) (HDB)
	.9100±.0685	.9800±.0151	Random forest

Persistence diagrams for this dataset were computed for each protein by considering the 3-D structure [provided in wwPDB consortium (2018)] as a point cloud in ${\mathbb{R}}^{3}$. This point cloud was built using the *x*, *y* and *z* position of each atom in the molecule at hand. With this information the persistent 0-dimensional and 1-dimensional homology is computed using Ripser from Tralie et al. (2018).

### 4.4 MPEG7

The mpeg-7 dataset from Bai et al. (2009) is a database of object shapes in black and white, with 1,400 shapes in 70 classes. An example from the original dataset is shown in Figure 4 along with the contour as described below.

To compute persistence diagrams, first the image contour is computed by placing observations from the point cloud into a sequence. The distance curve is computed as the distance from the center of the sequence. Sublevel set persistence is taken using the computed distance curves as point cloud data. Persistence diagrams for both 0-dimensional and 1-dimensional homology were computed for this dataset.

We used this dataset for a timing comparison of featurization methods from persistence diagrams. We do not report on the results of this dataset. An example notebook of MPEG7 is provided using only four shapes—apple, children, dog, and bone. This approach is due to the initial difficulty in getting accurate models for the full dataset. Due to the small number of samples (80 total) and lack of repeated sampling, the estimates provided for this dataset are not stable and are not reported.

### 4.5 HAM10000

The HAM10000 dataset provided by Philipp Tschandl et al. (2018) is a collection of 10,000 images of skin lesions with one 7 potential classifications: Actinic Keratoses and Intraepithelial Carcionma, Basal cell carcinoma, Benign keratosis, Dermatofibroma, Melanocytic nevi, Melanoma, Vascular skin lesions. A total of 18 persistence diagrams for this set were calculated using the methods outlined in Chung et al. (2018), 9 corresponding to the 0-dimensional homology and 9 corresponding to the 1-dimensional homology.

**FIGURE 9:**
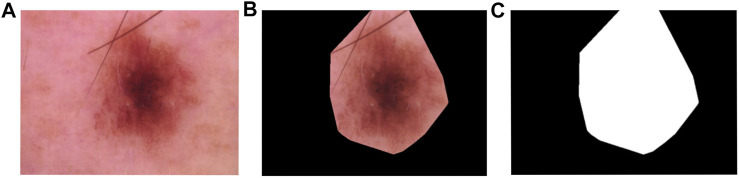
**(A)** Example of skin lesion in HAM10000 **(B)** Skin lesion with mask **(C)** Mask only dataset.

To obtain such diagrams, first a mask is computed by implementing the methodology proposed in Chung et al. (2018). In general terms, this method creates a filtration of binary images obtained from different thresholds to convert the gray scale image into a binary one. Once this binary filtration is obtained, the center most region of the image is computed using the “persistence” of each point in the binary filtration. An example image and this process is shown in Figure 9.

Once the mask is computed it is applied to the original image and then it is converted into three different color models: RGB, HSV , and XYZ. Each color model is split into their corresponding channels, and for each channel we use sublevel set filtration to obtain 0-dimensional and 1-dimensional persistence diagrams. In total, for each image on the data set we obtain 18 persistence diagrams, 9 in homological dimension 0 and 9 for homological dimension 1.

To tune the models, a random sample of 250 images were taken a ridge regression, random forest, and support vector machine model were fit to determine parameters. The remaining 9,750 images were split into an 80% training and 20% testing set to report final results.

To evaluate the HAM10000 dataset, due to the large number of birth and death pairs in each persistence diagram, subsampling of persistent features was required. Each observation in a data set, for example an image, will yield 18 persistence diagrams corresponding to homological features in that observation. In the HAM10000 dataset, there was an average of 5,783 birth-death pairs in each persistence diagram. This was an issue to complete computation for the vectorization methods, even for adaptive templates, so each persistence diagram was subsampled as follows.

The method of subsampling is two steps: Highly persistent features were always included, and a uniform random sampling method (without replacement) was used to sample the remaining points. The threshold for feature lifetime and number of points to sample was determined by using parameters that preserve the distribution of points in each persistence diagram. As a result, features in each persistence diagram with a lifetime of five or more were automatically included, and 5% of the rest of the points were also included. This resulted in sampled persistence diagrams with an average of 290 points each (Table 3).

**TABLE 3:** Characteristics of each dataset. The column headings can be explained as such: Observations—number of observations in the dataset, Diagrams—the number of homological types used to compute persistence diagrams, Average Pairs—the average number of birth/death pairs across the set of persistence diagrams for a single observation in the original dataset, and Min/Max Pairs—the minimum and maximum number of birth/death pairs across the set of persistence diagrams for a single observation in the original dataset.

Dataset characteristics				
Dataset	Observations	Diagrams	Average pairs	Min/Max pairs
MNIST	70,000	8	1.15	0/7
SHREC14	300	2	14	1/29
Protein	1,357	2	346	3/500
MPEG7	1,400	2	205	1/500
HAM10000	10,000	18	5,783	13/32610

## 5 User Guide

### 5.1 Available Functions

As part of the available code, a function for each method is included. Each function requires two sets of persistence diagrams, a training set and a testing set, and parameters specific to the function. The function returns two feature sets for that method, corresponding to the training and test set respectively. Each function also prints the time in seconds taken at the end of each run. In this section of the user guide each function is described, along with the required parameters for the function.

The **Multi-Scale Kernel** feature matrix can be computed using the function kernel_features or fast_kernel_features. It is recommended to use fast_kernel_features due to computation time. Both functions require a parameter sigma, denoted as s in the function with a default value of 4. In Reininghaus et al. (2015) this parameter is referred to as the scale parameter. From the closed form distribution of the Multi-Scale Kernel

$$k_{\sigma }\left(F,G\right)=\frac{1}{8\pi \sigma }\underset{p\in Fq\in G}{\sum }e^{-\frac{|\left|p-q\right|{|}^{2}}{8\sigma }}-e^{-\frac{|\left|p-\bar{q}\right|{|}^{2}}{8\sigma }},$$ (6)

we note that as sigma, *σ*, increases the function decreases. Increasing sigma results in a less diffuse kernel matrix, while decreasing sigma results in a more diffuse kernel matrix.

Due to time required for the Multi-Scale Kernel, there are two additional sets of functions that use Numba (Siu Kwan Lam and Seibert, 2015) for significantly faster computation. In the current implementation, these are not able to be combined with multi-core processing (MPI for example), and have a different format than the other functions included. These functions are provided in the github repository for this project, and were used to compute results for the Multi-Scale Kernel for the MNIST dataset.

The **Persistence Landscapes** features can be computed using the function landscape_features. The Multi-Scale Kernel function, landscape_features requires two parameters: the number of landscapes, n and resolution, r. The number of landscapes parameter, n, controls the number of landscapes used, and the resolution, r, controls the number of samples used to compute the landscapes. The default parameters for *n* is 5 and *r* is 100.

The **Persistence Images** can be computed using the function persistence_image_features. The persistence_image_features function requires two parameters, pixels and spread. The pixels, *p* is a pair that defines the number of pixels along each axis. The spread, *s*, is the standard deviation of the gaussian kernel used to generate the persistence image. It is worth noting that the implementation here uses the gaussian kernel, however, other distributions could be chosen so that s would correspond to parameters specific to the chosen distribution. Additionally, the weighting function is constant for this implementation. Increasing spread increases the variance for each distribution, resulting in larger “hot spots”. Increasing pixels provides a smoother distribution, whereas decreasing pixels yields a less smooth distribution. Note that increasing pixels increases computation time. This is demonstrated in Figure 7 in the methods section.

The **Adcock-Carlsson Coordinates** features can be computed using the function carlsson_coordinates, does not require any parameters. This function returns four different features for every type of persistence diagram provided. So for datasets that have persistence diagrams corresponding to 0-dimensional and 1-dimensional homology, 8 features are returned for machine learning. The features returned correspond to the four coordinates calculated in Adcock et al. (2016), and are:

$$\underset{i}{\sum }x_{i}\left(y_{i}-x_{i}\right),$$

$$\underset{i}{\sum }\left(y_{max}-y_{i}\right)\left(y_{i}-x_{i}\right)$$

$$\underset{i}{\sum }x_{i}^{2}{\left(y_{i}-x_{i}\right)}^{4},$$

$$\underset{i}{\sum }{\left(y_{max}-y_{i}\right)}^{2}{\left(y_{i}-x_{i}\right)}^{4}$$

The **Template Systems** features can be computed using the function tent_features, and has a choice of two parameters: d, which defines the number of bins in each axis and padding, which controls the padding around the collection of persistence diagrams. This function returns a training and testing set. This function computes the tent features from Perea et al. (2019).

The **Adaptive Template Systems** features can be called with the function adaptive_features, and requires the labels for the training set. Users can choose three different types of Adaptive Templates: Gaussian Mixture Modeling (GMM), Cover-Tree Entropy Reduction (CDER), and Hierarchical density-based clustering of applications with noise (HDBSCAN). The parameter d refers to the number of components when using the GMM model type. This would be minimally the number of classes in your data, and ideally represents closer to the number of distributions in the data that correspond to each observation. Details on these methods can be found in Polanco and Perea (2019a), as well as the original references linked in the methods section. During this evaluation, we evaluated adaptive templates using both GMM and CDER methods, but did not formally evaluate HDBSCAN. HDBSCAN was difficult to formally assess as we had difficulty with completion of the algorithm for some datasets. For those datasets we were able to complete, we did not notice an improvement over other adaptive methods.

## 6 Results

One consideration we must make before analysing the results comes from the computation of **Multi-Scale Kernel** features. As explained for each dataset in Section 4, more often than not we will compute multiple persistent diagrams per data point in a given data set. Such persistent diagram correspond to 0-dimensional, 1-dimensional, and in some cases 2-dimensional persistent homology (see details in Section 2). To compute Multi-Scale Kernel as given by Eq. 6 we require pairs of persistent diagrams. Since this multi-scale kernel provides a notion of similarity between persistent diagrams (Reininghaus et al., 2015) we require it to be computed between diagrams corresponding to the same dimension homology and method type. For example, the kernel matrix that corresponds to the 0-dimensional homology of a data set is computed using the persistence scale space kernel between two sets of persistence diagrams that represent the 0-dimensional homology. This means that for a dataset that has sets of 0-dimensional homology persistence diagrams and 1-dimensional homology persistence diagrams, two kernel matrices were returned (one per each dimension).

The kernel matrix used in our models is the sum of available kernels, and differs based on the persistence diagrams available for each dataset. While this does improve accuracy significantly over individual kernel matrices, other methods of combining kernel features were not explored in this paper, but is available in Gönen and Alpaydin (2011) for the interested reader. The available parameter, sigma, is consistent across all types of diagrams for our evaluation.

For each of the other methods, **Persistence Landscapes**, **Persistence Images**, **Adcock-Carlsson Coordinates**, **Template Systems**, and **Adaptive Template Systems**, each feature matrix was constructed for the relevant set of diagrams, and all topological types were used in fitting the same model.

The datasets used in this analysis were of varying size, both in terms of observations and the size of sets of persistence diagrams. As noted in the descriptions of data, the types of persistence diagrams calculated also differs. A summary of characteristics for each dataset is included in Table 3.

### 6.1 MNIST

The Multi-Scale Kernel features calculated yielded eight different kernel matrices, and the final kernel matrix was calculated using the unweighted summation of these kernels as in Gönen and Alpaydin (2011). Due to the time needed for computation of the Multi-Scale Kernel, a smaller set of 12,000 observations was used to report final results and a version of the kernel computation using Numba with a gpu target was necessary.

Table 4 shows complete results for the MNIST analysis. Four different methods (highlighted on the table) provided similar results for the MNIST dataset, and we note the SVM model had higher accuracy in each case. This table, and all subsequent results tables, include the method used to construct topological features, training and test accuracy, and model and parameters used for evaluation.

**TABLE 4:** Results from the MNIST Dataset using the average model classification accuracy ± standard deviation over 10 trials.

Full results for MNIST dataset			
Topological method	Training accuracy	Testing accuracy	Model type
Multi-scale kernel (sum of kernels for 12,000 observations)	.6895±.0035	.6932±.0117	SVM
Persistence landscapes	.8844±.0004	.8786±.0019	Ridge regression
	.9231±.0004	.9180±.0018	SVM (RBF)
	.5814±.0098	.5828±.0098	Random forest
Persistent images	.8997±.0005	.8934±.0021	Ridge regression
	.9368±.0004	.9199±.0023	**SVM (RBF)**
	.6889±.0036	.6953±.0123	Random forest
Adcock-carlsson coordinates	.8590±.0010	.8547±.0030	Ridge regression
	.9525±.0004	.9356±.0018	**SVM (RBF)**
	.7214±.0092	.7170±.0097	Random forest
Template systems	.896±.0005	.8959±.0017	Ridge regression
	.9638±.0003	.9477±.0015	**SVM (RBF)**
	.6967±.0035	.6973±.0031	Random forest
Adaptive template systems	.8819±.0016	.8817±.0027	Ridge regression (GMM)
	.9515±.0021	.9363±.0021	**SVM (RBF) (GMM)**
	.6914±.0188	.6932±.0209	Random forest

### 6.2 SHREC14

Results are reported in Table 1. Adaptive Template Systems and Persistence Landscapes were the two methods with highest classification accuracy on the test dataset, with Template Systems and the Multi-Scale Kernel performing nearly as well.

### 6.3 Protein Classification

**FIGURE 10:**
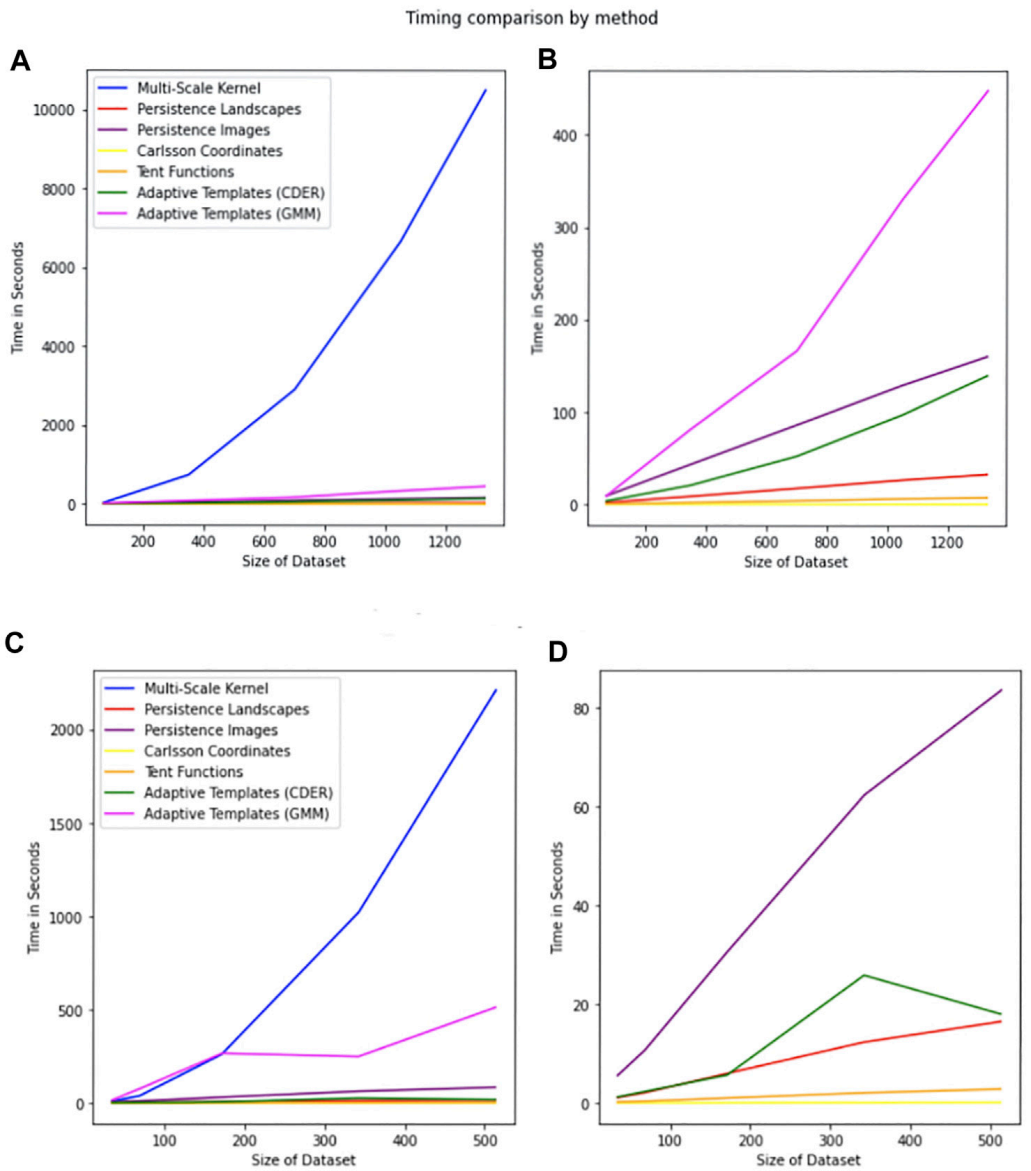
Comparison of timing by method. The legend is the same for all plots. The x-axis represents the size of the dataset, and the y-axis represents the time in seconds required for calculation of all of the persistence diagrams associated with the dataset of the given size. **(A)** Timings for the MPEG7 Dataset including the Multi-Scale Kernel. **(B)** Timings for the MPEG7 Dataset excluding the Multi-Scale Kernel. **(C)** Timings for the Protein Dataset including all features. **(D)** Timings for the Protein Dataset excluding Multi-Scale Kernel and Adaptive Templates (GMM).

Nearly all of the topological methods in this paper provided similar classification accuracy for this dataset. We observe the testing accuracy as higher than the training accuracy for this dataset, and the results are similar to those in Polanco and Perea (2019b). The Multi-Scale Kernel though did not perform as well and as shown in Figure 10 is the most computationally intensive. Results are reported in Table 2.

### 6.4 HAM10000

Due to run time for the large number of points in each persistence diagram, even after subsampling, results were not reported for the Multi-Scale Kernel or Adaptive Template Systems.

Results are listed in Table 5. The HAM10000 dataset presented the largest computational challenge during this review, and is a continued area of research.

**TABLE 5:** Results from the HAM10000 Dataset using the average model classification accuracy ± standard deviation over 10 trials.

Results for HAM10000 dataset			
Topological method	Training accuracy	Testing accuracy	Model type
Multi-scale kernel	Did not run		
Persistence landscapes	.8347±.0022	.6881±.0074	Ridge regression
	.6695±0	.6692±1.2e−16	SVM (RBF, *c* = 1)
	.6695±0	.6692±1.2e−16	Random forest
Persistent images (pixels = 20, spread = 1)	.7417±.0017	.6371±.0671	Ridge regression
	.7122±.0012	.6895±.0031	SVM (RBF, *c* = 1)
	.6695±0	.6692±1.2e−16	Random forest
Adcock-carlsson coordinates	.6719±.0007	.6696±.0025	Ridge regression
	.6801±.0009	.6710±.0019	SVM (RBF)
	.6695±0	.6692±1.12e−16	Random forest
Template systems (*d* = 10, *p* = 1.5)	.7193±.0015	.6987±.0041	Ridge regression
	.7830±.0024	.7303±.0054	SVM (RBF, *c* = 5)
	.6695±0	.6692±1.2e−16	Random forest
Adaptive template systems	Did not run		

## 7 Computation Time of Features

Formal timings were captured for all features for the 0-dimensional persistence diagrams for the MPEG7 and Protein Datasets. A comparison of timings is in Figure 10. The timing reported is for the generation of features from one type of persistence diagram for a dataset of that size. This means when computing a training feature set and testing feature set for multiple types of persistence diagrams, the expected time to generate features can be significantly longer. For example, in the MNIST dataset we compute four different types of persistence diagrams with both 0-dimensional and 1-dimensional homology, giving eight sets of features that can be generated for the sets of persistence diagrams for that dataset. Specific to the multi-scale kernel method, the timing reported is for a symmetric feature matrix that is nxn, where *n* is the number of observations in the dataset. This means the training feature set requires less computation time than a testing feature set of comparable size.

Additionally, during the review of these methods, we did not encounter significant issues with model fitting, hence formal timings were not recorded for this portion of the analysis. Conclusions from these timings are addressed in the discussion section.

### 7.1 Data Availability

The datasets, persistence diagrams (or code to compute the diagrams), and all other associated code for this study can be found in the machine learning methods for persistence diagrams github repository https://github.com/barnesd8/machine_learning_for_persistence.

For each of the five datasets, the following code is available:

• A jupyter notebook that loads and formats the persistence diagrams including images and does a preliminary model fitting on a subset of the data
• A python script that calculates the persistence diagrams from the original dataset - some of these are written using MPI depending on the size of the dataset
• A python script that fits models for random samples of the data to get mean estimates of accuracy for both the training and test dataset

These scripts reference modules included in the github repository, including a persistence methods script that calculates features from persistence diagrams for a training and test dataset. This uses a combination of other available functions and functions written specifically for this paper.

The **Template Systems** and **Adaptive Template Systems** methods use functions from https://github.com/lucho8908/adaptive_template_systems, which is the corresponding code repository for Polanco and Perea (2019a). The available methods in our extension include Tent Functions and Adaptive Template Functions (GMM, CDER, and HDBScan methods).

The **Adcock-Carlsson Coordinates** method is a function developed specifically for this paper, and includes the calculation of the 4 different features used in our analysis. The **Persistence Landscape** method uses the persistence landscape calculation from the Gudhi Package Dlotko (2021). The **Multi-Scale Kernel Method** has two included implementations, one is from Nathaniel Saul (2019) and is slower to compute, while the other is a faster implementation that can be used on larger datasets. All of the results in this paper were reported using the implementation written specifically for this paper. The **Persistence Images** features are also from Nathaniel Saul (2019). Additionally, many functions from Pedregosa et al. (2011) are used throughout.

## 8 Discussion

Adcock-Carlsson Coordinates, Tent Functions, and Persistence Landscapes scale well, and perform well even for large datasets. It is of note though that parameter choice will affect computation time. This was especially notable in the Template Features (Tent Functions) computation time. As the number of tent functions is increased, the time to compute features also increases. We observed a superlinear increase, however, even with this increase computation time was not a barrier for analysis.

Persistence Images and Adaptive Template Functions do not scale or perform as well, however, do provide good featurizations for accurate models and should be considered depending on the dataset. Specifically, the Adaptive Template Functions was not completed for the full HAM10000 dataset due to computation time.

When using these methods, it should be of note that the Multi-Scale Kernel method is computationally intensive, and does not scale well. Additionally, the accuracy achieved is not better than other methods for the datasets in this paper.

## 9 Conclusion

This paper reviews six methods for topological feature extraction for use in machine-learning algorithms. Persistence Landscapes, Adcock-Carlsson Coordinates, Template Systems, and Adaptive Template Systems perform consistently with minimal differences between datasets and types of persistence diagrams. These methods are also less expensive in terms of execution time. A main contribution of this paper is the availability of datasets, persistence diagrams, and code for others to use and contribute to the research community.
