# Supplementary figures and images for: A Comparative Study of Machine Learning Methods for Persistence Diagrams
Source: Front Artif Intell. 2021 Jul 28;4:681174. doi: 10.3389/frai.2021.681174 (PMC8355525; doi:10.3389/frai.2021.681174)

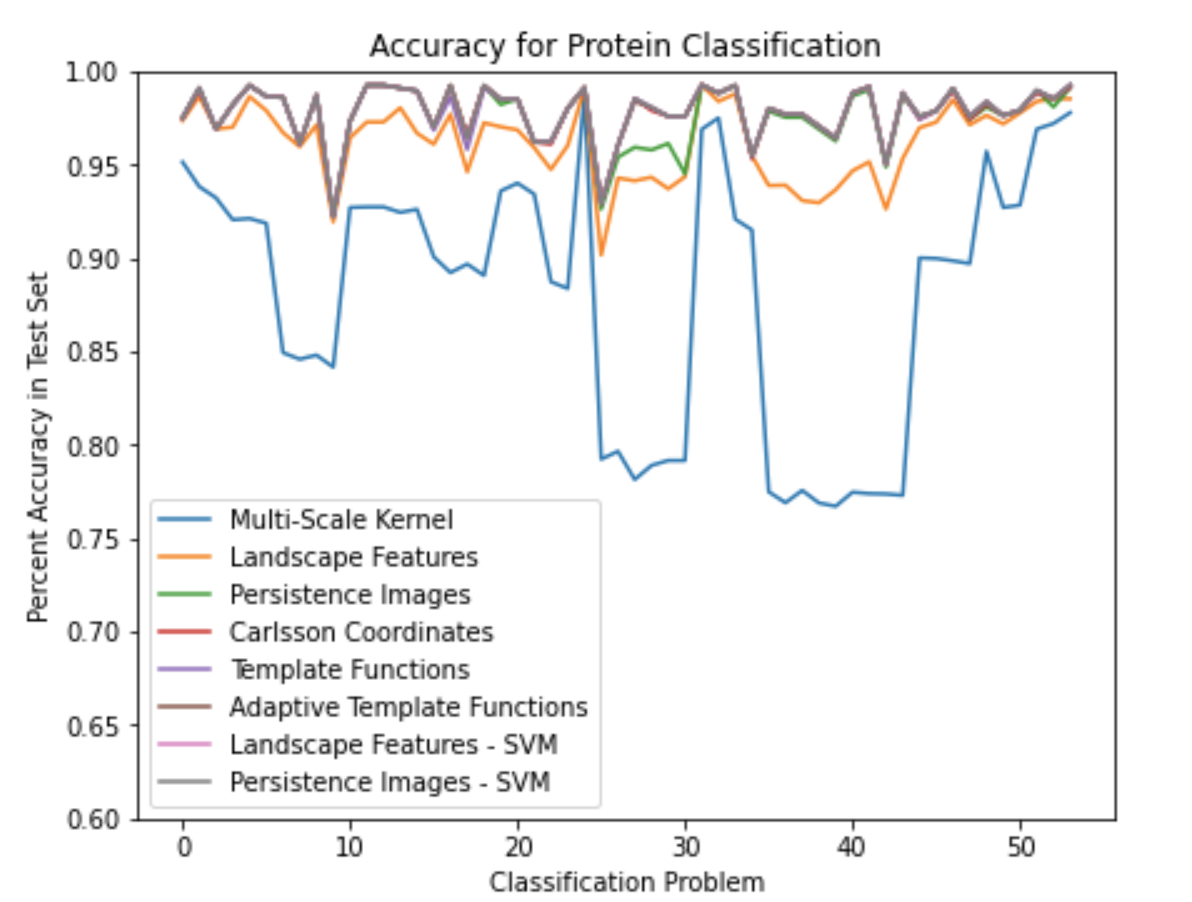

Supplement: Supplementary file 1 [file Image1.JPEG]
